# Intention to adopt electric transportation services by university students in emerging countries

**DOI:** 10.1371/journal.pone.0341736

**Published:** 2026-01-27

**Authors:** Diego Marcelo Cordero, Maria Fernanda Villavicencio, Kleber Antonio Luna, Yonimiler Castillo

**Affiliations:** 1 Department of Research, Academic Unit of Economic and Administrative Sciences, Catholic University of Cuenca, Bolívar, Cuenca, Ecuador; 2 Marketing Research, Academic Unit of Economic and Administrative Sciences, Catholic University of Cuenca, Bolívar, Cuenca, Ecuador; Vellore Institute of Technology, INDIA

## Abstract

This study analyzed the factors influencing university students’ intention to use electric bus services to commute to campuses in developing countries, specifically in Ecuador, Peru, and Colombia. A technology adoption model was proposed that integrates variables from the Technology Acceptance Model (TAM), Theory of Planned Behavior (TPB), and Unified Theory of Acceptance and Use of Technology (UTAUT), which helps in understanding, predicting, and explaining the acceptance and use of technologies, thus facilitating the design of strategies to improve adoption in various contexts. Data were collected from 1,158 students across different academic fields, genders, and academic levels in the countries studied. The analysis was conducted using structural equation modeling with the partial least squares technique. The key factors assessed included attitudes toward electric vehicles, perceived risk, consumer characteristics, emotions, hyperbolic discounting, intention to use electric transport, and social influence. The findings provide valuable insights into the determinants of electric transportation service adoption among university students and offer guidance for implementing sustainable transportation solutions in educational institutions in developing countries.

## 1. Introduction

The transition to electric transportation, particularly the adoption of electric buses, presents a significant opportunity for sustainable urban mobility [1]. This shift addresses environmental concerns and offers both economic and social benefits [2]. One of the primary advantages of electric buses is the reduction of greenhouse gas emissions [3].Traditional fuel oil buses significantly contribute to urban air pollution and carbon emissions [4]. Studies have shown that switching to electric buses can substantially decrease these emissions, contributing to cleaner air and improved public health [5]. However, electric buses are quieter than their fuel-oil counterparts, reducing noise pollution in urban areas [6]. Economically, electric buses offer long-term savings [7]. Although the initial investment in electric buses and the necessary charging infrastructure can be high, the total cost of ownership tends to be lower because of reduced fuel and maintenance costs [8]. Electric buses have fewer moving parts than diesel buses, which translates to lower maintenance requirements and costs over their lifetimes [9].

According to Garrigosa [10], each electric bus results in an annual reduction of 88 tons of CO2 emissions and savings of 33,000 liters of fuel. The U.S. The Department of Transportation [11] states that over a 12-year lifespan, a zero-emission bus can prevent up to 1,690 tons of CO2 emissions. Regarding noise levels, a study by the C40 Cities Finance Facility (CFF) in collaboration with Grütter Consulting [12] for the Eje 8 Sur corridor in Mexico City found a 50% reduction in noise levels compared with diesel buses. Regarding costs, the My Transport project of the German Cooperation (GIZ), led by Kruse [13] in Costa Rica, showed that the average daily fuel cost for a diesel bus was six times higher than that for an electric bus. Macián et al. [14] found, in a study for Valencia, that electric buses consume 30% of the total energy consumed by a diesel engine under urban conditions, demonstrating savings in terms of energy efficiency.

From a social perspective, the adoption of electric buses can improve urban mobility and accessibility [15]. Electric buses can provide reliable and efficient public transportation, which is essential for connecting underserved communities to economic opportunities and essential services. This is particularly important in rapidly growing urban areas in developing countries, where transportation infrastructure often lags population growth [16]. Policy support is crucial to the widespread adoption of electric buses. Government incentives, such as subsidies for purchasing electric buses and investments in charging infrastructure, can accelerate this transition [17]. Furthermore, various policy measures can support the deployment of electric buses. These include financial incentives, such as subsidies and grants; regulatory measures, such as emission standards and zero-emission zones; and supporting infrastructure development, particularly charging stations [18]. Innovative financing models and public-private partnerships can play a vital role in overcoming the financial barriers associated with the initial deployment of electric buses [19] Programs that focus on equity, ensuring that low-income and disadvantaged communities also benefit from cleaner transportation options, are essential for inclusive sustainable development [20].

The adoption of electric vehicles (EVs) by university students is gaining traction as a sustainable transportation solution. This trend is driven by various factors, including environmental awareness, economic benefits, and institutional support [21]. One significant factor influencing students’ electric vehicle (EV) adoption is their growing environmental consciousness [22]. University students are becoming increasingly aware of the environmental impacts of their transportation choices and are more likely to choose EVs to reduce their carbon footprint [23]. This aligns with the broader societal trend towards sustainability and the increasing importance of addressing climate change. Institutional support from universities can further encourage EV adoption. Many universities have implemented policies and infrastructures to support sustainable transportation [24].

Universities offering EV charging stations, parking incentives for EV owners, and incorporating EVs into campus fleets have significantly boosted student adoption rates. These initiatives not only facilitate the use of EVs but also promote a culture of sustainability on campus [25]. Moreover, the availability of information and education on the benefits of EVs is crucial. Universities play a vital role in disseminating knowledge and fostering communities that value sustainable practices [26]. Students educated on the environmental and economic advantages of EVs are more likely to consider adopting electric bus services.

Ecuador, Colombia, and Peru are developing countries in South America with emerging economies that are rich in natural resources, but face social, political, and economic challenges in their growth process [27]. Although they face significant economic and social challenges, they promote environmental policies to protect their ecosystems and renewable energy, conserve biodiversity, and address climate change [28].

Through the initiatives of academic authorities from various universities in the countries under study, electric transportation services have been implemented to transport students from different programmers to university campuses. Therefore, analysis of the intention to use this transportation system is necessary to provide valuable information on the perceptions, motivations, and barriers faced by potential users. This initiative aligns with the energy efficiency laws in Ecuador, Colombia, and Peru, which aim to promote efficient energy use, reduce emissions, and encourage renewable energy use [29].

Ecuador, Colombia, and Peru were selected for this study as developing South American countries with emerging economies that promote environmental policies despite facing socioeconomic and political challenges [30]. Shared cultural contexts influence similar responses to the adoption of technology, although regional differences may arise due to factors such as education, employment, and urban residence [31].

While age plays a key role in the choice of environmentally friendly products and services, the centennial generation is more globally aware and concerned about its role in improving the world than other generations [32,33]. However, several studies have shown significant differences between generations in the use of technology, with younger generations showing higher adoption rates [34]. Therefore, the adoption of EVs by university students is gaining traction as a sustainable transportation solution.

The results of the study in Ecuador, Peru, and Colombia allow us to draw relevant conclusions about university students and can be generalized to other Latin American countries due to socioeconomic, cultural, and academic similarities [35]. The homogeneity of this young and connected population strengthens this generalization, although it is acknowledged that local variations may exist, which could require specific adjustments in future research [36].

Despite advances in the implementation of electric buses at universities in these countries, there remains a gap in knowledge regarding students’ intention to adopt this service, particularly in multinational studies and in the context of student collective transportation. To address this gap, the study integrates the TAM, TPB, and UTAUT models, overcoming the limitations of individual models by considering attitudes, social norms, perceived control, and performance expectations. In practice, its findings can help optimize university transportation, reduce costs, and promote sustainability, while also guiding policies and incentives to increase adoption.

The main objective of this research is to determine the factors influencing university students’ intention to adopt electric bus services, in Ecuador, Colombia, and Peru. The specific objectives are to analyze the impact of attitude, social norms, and perceived control on the intention to adopt using an integrated TAM-TPB-UTAUT model, comparing differences and similarities among the three countries, assessing the relevance of university policies, charging infrastructure, and environmental awareness, and providing practical recommendations to optimize electric bus services and promote student sustainability. To achieve this, the study reviews relevant literature on technology adoption models, selecting TAM, TPB, and UTAUT as the framework to guide students’ intention to use electric bus services. Based on this framework, the following research questions were formulated: What variables influence university students’ decision to adopt an electric bus service? What is the appropriate model for reflecting the influence of these variables on adoption?

## 2. Theoretical Background

### 2.1. Technology adoption models

Technology adoption models seek to explain how and why individuals and organizations adopt and use new technologies. These models are crucial for understanding the process of acceptance and diffusion of technological innovations, as well as for identifying the factors that influence successful adoption.

The selection of TPB, TAM, and UTAUT variables to study the intention to use electric bus services was based on their ability to address psychological, technological, and social factors. These models provide insights into consumer intentions and perceptions and identify interventions to promote EV adoption by representing a new technology that consumers should perceive as beneficial and easy to integrate [37]. They also provide a structured framework for the analysis. The relevance of these models lies in providing a comprehensive view of the consumer decision process regarding EVs [38,39].

#### 2.1.1. Theory of reasoned action (TRA) and theory of planned behavior (TPB).

Matharu, Jain, and Kamboj [40] proposed that TPB is a valuable tool for understanding why consumers adopt or reject sustainable purchasing practices, serving as a conceptual framework for analyzing their intentions and actions, as intention is a focal construct and summarizing the strength of an individual’s motivation to perform an objective behavior [41]. Green purchasing behavior was first addressed in the TRA which is based on the premise that people are rational and make decisions based on available information [42,43] and later, due to its limitations, TPB was used to explain it. TRA and TPB are psychological models that explain how attitudes and intentions influence human behavior.

Ajzen and Fishbein [42], suggest that a person’s behavior is determined by his or her intention to perform that behavior, which in turn is influenced by two main factors: attitudes, which are the positive or negative evaluation an individual has towards the behavior in question, and subjective norms, which represent the perception of the social pressure a person feels to perform or not perform the behavior. According to Ku’zniar et al. [44], the relationship between individual attitudes and behavior is a central focus of consumer behavior research. According to the TPB, attitude emerges as a key predictor of behavioral intentions, including purchase decisions [45].

However, TPB, developed by Ajzen [45] as an extension of TRA, incorporates a third component: perceived behavioral control, which refers to a person’s perception of his or her ability to perform a behavior, and may include internal factors (such as skills) and external factors (such as resources or barriers) [46]. The TPB argues that in addition to attitudes and subjective norms, perceived behavioral control also influences the intention to perform a behavior, which in turn affects actual behavior. This theory is especially useful for understanding behavior in contexts where people may feel that they do not have full control over their actions, as in the case of purchasing green or sustainable products [47].

#### 2.1.2. Technology acceptance model (TAM) and unified theory of acceptance and use of technology (UTAUT).

The literature proposes a wide range of models to explain IT acceptance. Yaseen and El Qirem [48] conducted a systematic review of some of these models, including the theory of reasoned action, theory of planned behavior, technology acceptance model, and social cognitive theory. Although each model provides valuable insights, the UTAUT proposed by Venkatesh et al. [49] has emerged as a unified theoretical framework that integrates the key constructs of these models [50]. Existing studies show that this model, although originally designed for other contexts, can be adapted to analyze people’s intentions to use emerging technologies. Researchers are exploring new constructs and relationships and the UTAUT model has shown promise for studying the adoption of autonomous vehicles, although its application in this field is still in its early stages [38].

UTAUT is a model that incorporates several TAM factors and has been widely applied in various technological contexts, postulating that users’ perception of the usefulness and ease of use of a system directly influences their attitudes and intention to use it and, ultimately, its effective adoption [51], considering that the attitude construct refers to the extent to which individuals are willing to accept a new technology, such as EVs [52]. Other variables that can influence the adoption of new technologies, such as social influence, can be summarized as the environmental pressures exerted by others on individuals when they decide to adopt or avoid a behavior [52]. The TRA contends that an individual’s behavioral intentions are affected by social norms and attitudes [53].

Perceived risk is a term associated with potential losses that customers consider when purchasing or choosing a product or service. Consequently, the risks perceived by consumers in the context of EVs include high costs, autonomy, and charging infrastructure [54]. Furthermore, Tiwari, Tandon, and Mittal [55] add that environmental concerns, perceptions of behavioral control, and economic incentives are equally relevant.

Hyperbolic discounting was added as a variable in the TPB by identifying the general gap between behavioral intention and actual behavior [56]. Several studies based on theories such as TPB and TAM [57–60] have shown that individual characteristics exert a significant influence on the formation of attitudes and risk perceptions towards new technologies [61].

The integration of multiple theories in a study allows us to capture the complexity of the phenomenon and explore various dimensions of the behavior under investigation [38]. Although some variables may be correlated, each contributes unique value to the model. These models provide insights into consumer intentions and perceptions and identify interventions to promote EV adoption by representing a new technology that consumers should perceive as beneficial and easy to integrate [37]. They also provide a structured framework for the analysis. The relevance of these models lies in providing a comprehensive view of the consumer decision process regarding EVs [39].

Hyperbolic discounting, manifested through options such as installment payments, fixed discounts, profit sharing, rewards, and profit delays (including EV incentives), exerts a positive influence on consumers’ vehicle (EV) purchase decisions. [56] found that consumers’ environmental concerns translate into higher purchase intention and, subsequently, higher EV adoption. Furthermore, it has been suggested that an increase in the magnitude of the hyperbolic discount could intensify the positive connection between purchase intention and actual adoption of these vehicles [58,60].

According to [60], several studies have consistently shown that psychological factors such as experience, attitude, perceived behavioral control, emotions, social influence, personal moral norms, symbols, and hyperbolic discounting are significant variables for EV adoption [62].

### 2.2. Variable selection

The variables included in this study were selected to develop a comprehensive framework that captures the complexity of electric bus adoption in developing countries. Traditional models such as TAM, TPB, and UTAUT emphasize cognitive and psychological factors, but their isolated use is insufficient to fully explain adoption behaviors in emerging economies, where social, contextual, and temporal factors are also important. To address this, variables were chosen based on their relevance to individual, social, and behavioral dimensions. Key constructs such as attitude toward EVs, perceived risk, emotions, social influence, and intention to use electric transportation are derived from TAM, TPB, and UTAUT, while consumer characteristics and hyperbolic discounting account for decision-making heterogeneity. These variables collectively strengthen the explanatory power of the framework by integrating cognitive, affective, and contextual determinants (see Table 1). This integration enhances theoretical coherence and practical applicability, supporting the design of strategies and policies to promote electric mobility in Latin American university contexts.

**Table 1:** Variables of study.

Variable	TAM	TPB	UTAUT	References
Emotions (EMO)		✓		[63–66]
Perceived risk (PRI)	✓	✓		[67,68]
Attitude towards electric vehicles (AEV)	✓	✓		[69,70]
Consumer characteristics (CCH)			✓	[69–72]
Intention to use electric transportation (IET)	✓	✓	✓	[73–75]
Hyperbolic discounting (HDI)		✓		[76–78]
Social influence (SOI)		✓	✓	[76,79–81]

### 2.3. Hypothesis development

The research hypothesis establishes a logical relationship between two or more variables expressed in an affirmative manner. The research hypotheses are presented in the following section. To do this, each variable was analyzed, theoretically justifying the relationship between them. At the end of each section, we formulated a specific hypothesis.

#### 2.3.1. Emotions (EMO).

Positive anticipated emotion (POE) has the greatest impact on electric vehicle purchase intention, followed by attitude, indicating that emotions (EMO) positively influence attitudes towards electric vehicles [63,64]. Anticipated positive emotions, particularly those related to the green aspect of battery electric cars (BECs) and supportive government policies, significantly influence consumer attitudes towards EV purchases in emerging markets [82].

Explicit emotional attitudes towards electric cars were positive compared to petrol cars, but implicit attitudes did not show the same positivity, indicating a nuanced relationship between emotions (EMO) and attitudes towards electric vehicles [83]. Emotions positively influence consumer adoption of EVs through the mediating effect of perceived value, as indicated in research on emotions (EMO) and EV adoption behavior [65].

Consumer emotions (EMO), specifically satisfaction and trust, positively influence attitudes toward EVs, which impacts purchase intention [66]. Emotions play an important role in shaping consumer preferences for EVs as they are influenced by unique design features that appeal to emotions and ultimately positively impact attitudes [84]. Therefore, we propose the following hypothesis: H1. Emotions (EMO) positively influence attitudes towards electric vehicles (AEV)

#### 2.3.2. Perceived risk (PRI).

Emotions (EMO) positively influence the perception of the risk of acquiring EVs, with negative emotions predominating, especially when it comes to time-related issues such as charging and maintenance [85]. Emotions (EMO) as an EV driving experience negatively affects perceived risk in the context of EV adoption [86].

Emotions play an important role in influencing risk perception and adjusting responses to environmental factors, as described in the theories of valuation bias and feelings of information [67]. While emotions play an important role in shaping consumer attitudes and purchasing intentions towards EVs [63], the relationship between emotions and perceived risk is more nuanced, and positive anticipated emotions have a greater impact on purchase intentions than negative anticipated emotions [63].

Therefore, although emotions can influence consumer behavior and attitudes toward EVs, they may not always correlate directly with increased risk perception, as emotions can affect consumer perception in various ways [68]. Thus, we propose the following hypothesis: H2. Emotions (EMO) positively influence perceived risk (PRI).

#### 2.3.3. Attitude towards electric vehicles (AEV).

Hyperbolic discounts positively influence attitudes towards EVs, impacting consumer choices and purchasing behavior, as highlighted in a study on EV adoption in India [87]. Hyperbolic discounts on EVs positively influence attitudes towards EVs because price acceptance is an important factor influencing the intention to purchase these vehicles [88].

Consumers’ motivations to purchase EVs revealed that profit motives, which could include hyperbolic discounts, were important predictors of EV purchase behavior, indicating a positive correlation between profit motives and purchase intentions [89]. Consumer attitudes towards EVs stress the importance of socioeconomic factors in influencing buyer decisions and suggest that incentives such as hyperbolic discounts could play a decisive role in promoting EV adoption [69]. Therefore, offering hyperbolic discounts on the purchase of EVs can have a positive impact on attitudes towards EVs, which could lead to increased adoption rates [70]. It is based on the idea that individual preferences for immediate rewards can influence the perception of the short-term benefits of a technology, the following hypothesis is proposed: H3. Hyperbolic discounting (HDI) positively influences attitudes towards electric vehicles (AEV).

#### 2.3.4. Consumer characteristics (CCH).

Consumer characteristics, such as income, age, and education, significantly shaped attitudes toward EVs as well as attitude, perceived control, norms, and concern for the environment. key that affects the adoption of this type of vehicles [71]. Consumer characteristics, such as lifestyle and ostentatious consumption, positively influence attitudes towards EVs through functional and nonfunctional values, which impact purchasing intentions and ecological behavior [69]. Consumer characteristics, specifically gender, age, and income, influence attitudes towards EVs, while females show a positive influence of environmental concern on attitudes towards EVs. Income level influences purchase intent [72], whereas consumer awareness, perceived risk, and functional characteristics significantly influence attitudes towards EVs, thereby reducing post-purchase dissonance [90]. In addition, a study of consumer motives influencing EV purchase behavior indicated that personal moral norms have a positive impact on the emotions associated with EV purchases, further emphasizing the role of consumer characteristics in influencing purchasing attitudes and intentions [91].

These insights underscore the need for policymakers and marketers to consider consumer characteristics when developing strategies to promote EV adoption and address environmental challenges [91]. Individual characteristics can influence people’s attitudes and risk perceptions, as proposed by the TPB, TAM, and UTAUT and based on the idea that individual characteristics can influence people’s attitudes and risk perceptions, as proposed by the TPB and TAM. Thus, the following hypothesis is proposed: H4. The characteristics of the consumer (CCH) positively influence attitudes towards electric vehicles (AEV). H5. Consumer characteristics (CCH) positively influence perceived risk (PRI).

#### 2.3.5. Intention to use electric transportation (IET).

One of the decision criteria for adopting an electric car is perceived risk. Acquiring an EV is costly and risky; therefore, it is unlikely that typical consumers will make decisions without extensive reasoning [73]. This behavior underpins the TRA [92]. Factors that increase consumers’ perceived risk when purchasing an EV include limited battery-charging capacity, insufficient charging infrastructure, and costs [93]. Consumers’ perception of these risks negatively influences their intention to purchase electric cars [74,94].

Thøgersen and Ebsen [73] found that, in Denmark, the strongest predictor of intention to purchase EVs is a lack of trust, with a negative relationship. Additionally, perceived uncertainty has a detrimental effect on purchase intention. Therefore, the following hypothesis is formulated: H6: Perceived risk (PRI) negatively influences the intention to use electric transportation (IET). Various psychological theories have suggested that emotions can have a significant impact on people’s attitudes and perceptions. Principles of TAM and TPB. This is an extension of the TRA, which incorporates perceived behavioral control [45].

A study conducted in India by Deka, Dutta, Yazdanpanah, & Komendantova [95], demonstrated two constructs that explain the intention to purchase EVs: subjective norm and perceived behavioral control. However, this attitude was not significant in this study. Additionally, the study incorporated three constructs: cost, herd behavior, and personal norms. These were not individually significant; however, when cost reduction was combined with perceived behavioral control and attitude, people had a favorable attitude toward adopting EVs.

The study by Bobeth and Kastner [96], conducted in Germany, addresses the question of whether the decision to purchase an electric car is based on rational choice or normative behavior. The variables analyzed included the perceived usefulness and ease of use of EVs, intention to purchase an EV, and individual-related factors such as technical interest and usage experience. This study tested three action models: an adjusted TAM, an adjusted Norm Activation Model (NAM), and an integrative model that combines predictors from both models. The results revealed that, in the early stage of EV diffusion, perceived usefulness and ease of use may be influenced by individuals’ technical interest and usage experience. Additionally, significant relevance of social norms was identified in the EV adoption process, although it may be difficult to distinguish between descriptive and normative social norms.

Environmental awareness is another emotional element widely studied regarding EV use. This has a positive relationship with the intention to purchase EVs [97] and [98]. Therefore, the following hypothesis is proposed: H7: Emotions (EMO) positively influence the intention to use electric transportation (IET).

Attitudes towards EVs vary according to the behavior of different variables that influence purchase intention. Some of these are: the availability of nearby charging stations [99] positively influences; when the government offers subsidies, people have a favorable attitude toward acquiring EVs [100,101]; and explicit and implicit attitudes, personal norms, and perceived utility are significant predictors of the intention to purchase electric cars [102]. Another factor is the experience in using EVs; several studies indicate that people who have already used EVs have a direct relationship with the purchase of EVs [103]. Current EV owners show a greater tendency to acquire another EV than those who have vehicles of other fuel types [104]. However, this is not always true. Studies by [105] demonstrated this in Poland and Denmark.

In general, a favorable attitude towards electric cars increases consumers’ intention to purchase them [73]. In addition, attitude, subjective norms, and perceived behavioral control are significantly associated with purchasing EVs [106]. Based on the above, the following hypothesis was formulated: H8: Attitude towards electric vehicles (AEV) positively influences the intention to use electric transportation (IET).

Additionally, [107,108] found that a higher level of education was positively related to the intention to purchase EVs. Likewise, [109] highlights that marital status, especially marriage, positively increases the likelihood of acquiring EV. Therefore, the hypothesis was as follows: H9: Consumer characteristics (CCH) positively influence the intention to use electric transportation (IET).

#### 2.3.6. Hyperbolic discounting (HDI).

Financial incentives positively influence intention to purchase EVs [110]. However, other studies indicate that financial incentive policies do not significantly affect intention to adopt EVs [76]. In the case of [77], with data from 50 US states, it was found that the adoption rates of EVs respond more to changes in state environmental policies than to financial incentives.

Behavioral economists refer to individual preferences for small but immediate rewards over larger ones in future hyperbolic discounting [111]. Hyperbolic discounting functions are characterized by a relatively high discount rate over short horizons and a relatively low discount rate over long horizons [78]. This phenomenon has been observed in EV consumers who prefer small but immediate financial incentives when purchasing an electric car to larger incentives in the future [112]. Therefore, the following hypothesis is formulated: H10: Hyperbolic discounting (HDI) positively influences the intention to use electric transportation (IET).

#### 2.3.7. Social influence (SOI).

Hu, Chen, & Davison [79] showed that social factors can determine consumer purchasing behavior. Wang et al. [76] found that the cognitive and evaluative dimensions of social identity have a significant effect on purchasing behavior.

In the case of purchasing EVs, some authors have demonstrated that social influence is positively related to intention to buy EVs [80]. In a study of Chinese residents, Cui et al. [81] found that social influence was the fourth most significant predictor of EV sales. Accordingly, we hypothesize the following: H11: Social influence (SOI) positively influences intention to use electric transportation (IET).

**Fig 1:**
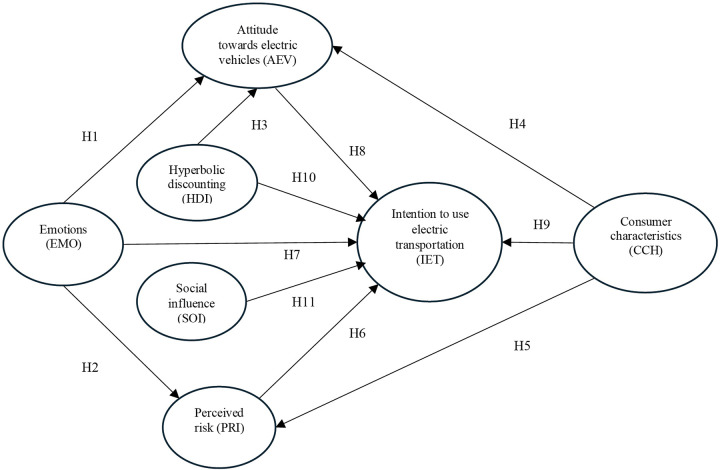
Research and hypothesis model.

Fig 1 presents the proposed model illustrating the intention to use electric transportation based on the 11 hypotheses developed from the theory. There is substantial evidence from studies on EV adoption that have employed nonlinear models such as artificial neural networks [113–115], decision trees [116], nonlinear logistic models [117], the innovation diffusion model (Bass Model) [118], and agent-based systems [119]. These models effectively identify complex relationships and threshold effects by considering factors such as past behavior, demographic characteristics, and network influences. However, these factors were not the primary focus of this study.

Given that this is an exploratory study conducted in an emerging country aimed at analyzing the factors influencing university students’ intention to use electric bus services, a linear model based on PLS-SEM was chosen. This selection is justified by its suitability for exploratory studies with limited data, ease of interpretation, and strong performance in contexts with non-normal data or small sample sizes [120,121]. In contrast, nonlinear models require larger samples and additional statistical assumptions [122] that do not apply in this case; therefore, because the objective of the study is to explain the factors affecting adoption, a linear model is the most appropriate choice [123,124]. If the goal is to predict behaviors and capture complex relationships, a nonlinear model is preferable [125,126].

## 3. Research methodology

### 3.1. Instrument

The questionnaire contained demographic information of the informants and indicators developed based on theoretical references and evaluated on a Likert scale from 1 to 5 with the following parameters: strongly disagree, disagree, neither agree nor disagree, agree, and strongly agree (see S1 Appendix). In PLS-SEM, it is recommended to use two–four items per variable to ensure reliability and validity. Some authors argue that a smaller number of items can be effective if they are well-designed and properly targeted [127,128]. Nonetheless, the possibility of expanding the number of items in future studies is acknowledged, as this could offer additional insights into the robustness and generalizability of the model.

### 3.2. Population and sample

This study did not involve the collection of sensitive data or conduct experiments with human participants. The data was obtained through anonymous and voluntary surveys designed solely for academic purposes and without any potential risks to the participants. Therefore, it was not considered necessary to obtain approval from an institutional ethics committee (IRB), as no identifiable personal data was collected, and no interventions were conducted.

Voluntary surveys ensure high participation rates, collect high-quality data, and enable participants to feel valued and safe. They encourage honest responses, especially on sensitive topics, which enhances the reliability of research findings [129,130]. The authors acknowledge the potential self-selection bias in voluntary surveys on the use of electric buses and justify their application because of logistical constraints. To mitigate this bias, the sample was diversified by distributing surveys across different contexts, schedules, and segments of university communities in the three countries. For future research, it is necessary to include control questions that allow for the assessment of participants’ prior knowledge levels and detection of possible biases in their responses.

A survey technique was employed to evaluate the proposed model, and an online questionnaire was designed using KoBoToolbox, targeting university students from accredited institutions in Ecuador, Colombia, and Peru. Informed consent was obtained from adult university students through online surveys, as participants were informed about the study’s objectives, potential risks, and confidentiality conditions. Informed consent was granted digitally, through the participants’ explicit acceptance upon accessing and completing the online survey, after having read the relevant information about the study’s objectives, risks, and confidentiality conditions. It is clarified that this was implicit digital informed consent, not verbal. It was not necessary to obtain consent from parents or guardians as the participants were of legal age. Consent was obtained indirectly from the survey system, which recorded the responses.

By virtue of being students, informants have access to the Internet both on university campuses and at home. Additionally, surveys were conducted in university environments, where Internet access is free and available to the entire student community, thus minimizing the risk of exclusion due to lack of connectivity [131]. Therefore, we consider that the potential bias from the overrepresentation of students with better internet access is low and does not significantly affect the validity of the results.

The sample size was determined based on the characteristics of the proposed model considering seven constructs, five of which had the most significant influence on the dependent variable. According to the Partial Least Squares (PLS) method, the minimum sample size was calculated by multiplying ten by the constructs affecting the dependent variable (10 ×  5 =  50). After performing an 80% power analysis, the required sample size was increased to 90 cases (50 + 40). However, 1,158 refined surveys have been conducted.

As observed in Table 2, according to the information provided by the higher education regulatory bodies of each country, a higher proportion of female students than male students are evident. Similarly, the percentages distributed by age group are representative. The total population consisted of 6,077,281 university students. By applying the sample estimation formula with a 95% confidence level and 5% margin of error, a representative sample was obtained for each country. The probabilistic sampling method ensures the representativeness of the population and allows researchers to make valid inferences about the study universe [132,133].

**Table 2:** University students by country.

Country	Number of University Students	Gender	Rate	Edad	Rate	Reporting Institution
Ecuador	887,171	Male Female	47% 53%	17-22 23-27 >28	72.20% 25.65% 2.15%	National Higher Education Information System
Perú	2,714,277	Male Female	49% 51%	17-22 23-27 >28	81.43% 16.61% 1.96%	National Institute of Statistics and Informatics
Colombia	2,475,833	Male Female	43.5% 56.5%	17-22 23-27 >28	78.74% 18.94% 2.32%	National Higher Education Information System

Ecuador, Peru, and Colombia share country branding strategies focused on tourism, culture, and exports as well as demographic, cultural, and social similarities [134]. These commonalities justify the representativeness of the university student sample as they share a sociocultural environment, ideology, biodiversity, and common challenges [135], with data gathered from April 26 to July 11, 2024. The selected period was based on the project’s research schedule. As this was a cross-sectional study, the questions asked reflected respondents’ perceptions at that specific moment. Therefore, the period did not influence the research results; the length of the period was irrelevant, because the unit of analysis was university students.

### 3.3. Data quality

Prior to conducting the PLS-SEM analysis, the dataset was rigorously cleaned to ensure high-quality and reliable data. Records with more than 10% missing information were removed, while cases with minor omissions were discarded without applying imputation techniques, considering the sufficiently large sample size (N = 1,158). No adjustments for multiple comparisons were performed, as the primary objective of the study was to evaluate an integrated structural model rather than test multiple independent hypotheses. These steps ensured that the data set used for analysis was both robust and appropriate for generating valid and interpretable results.

### 3.4. Demographic date

According to the collected information, 24.26% of the students were from Ecuador, 28.67% were from Peru, and 47.07% were from Colombia. Additionally, sociodemographic variables such as age and sex were included in the analysis. Table 3 shows the respondents’ demographic compositions, as explained in the previous paragraph.

**Table 3:** Demographic profile of the informant.

Category	Frequency	Percentage
Country:		
Ecuador	281	24.26%
Peru	332	28.67%
Colombia	545	47.07%
Age:		
17-22	897	77.46%
23-27	227	19.60%
>28	34	2.94%
Genre:		
Male	346	29.88%
Female	790	68.22%
LGBTI	22	1.90%

To assess the representativeness of the sample, demographic distributions were compared with the total university population in each country (Table 2). In terms of gender, the sample consisted of 29.88% male and 68.22% female students, which closely aligns with the population proportions in Ecuador (47% male, 53% female), Peru (49% male, 51% female), and Colombia (43.5% male, 56.5% female). Regarding age, 77.46% of the sample were aged 17–22, 19.60% aged 23–27, and 2.94% over 28, which is consistent with the population distribution in the three countries. These comparisons indicate that the sample is broadly representative of the university populations in Ecuador, Peru, and Colombia, supporting the validity of subsequent analyses. The PLS-SEM model is less dependent on strict sample randomness, and the large sample size (N = 1,158) provides sufficient statistical power to support the validity of the analyses.

### 3.5. Structural equation modeling approach (SEM)

In this study, Partial Least Squares Structural Equation Modeling (PLS-SEM) was employed due to its suitability for exploratory and predictive research, its ability to handle moderate sample sizes and non-normal data, and its flexibility in modeling both formative and reflective latent variables [136,137]. PLS-SEM is also robust against collinearity and allows the analysis of moderating effects and complex interactions [138,139]. In contrast, Covariance-Based SEM (CB-SEM) is primarily suited for theory confirmation and requires stricter assumptions regarding multivariate normality and larger sample sizes [140,141]. Potential nonlinear relationships and group-based analyses were conceptually considered to evaluate complex effects among the model’s variables; however, since the main objective of this study is to identify and assess the factors determining EV adoption intention, these analyses were not conducted and are proposed for future research to explore complex relationships and segment-specific effects.

Although PLS-SEM is appropriate for present research, future studies may consider CB-SEM to validate theoretical models [142], Bayesian SEM for more flexible estimations [143], or Nonlinear SEM to capture more complex relationships [144]. This approach ensures a comprehensive and robust understanding of electric bus adoption in emerging countries, while providing methodological guidance for subsequent research addressing nonlinear and group-specific effects.

## 4. Results

The model was analyzed using partial least squares (PLS) with Smart PLS 4.1.0.4 software, which considered the measurement model and structural model [145].

### 4.1. Measurement model

**Fig 2:**
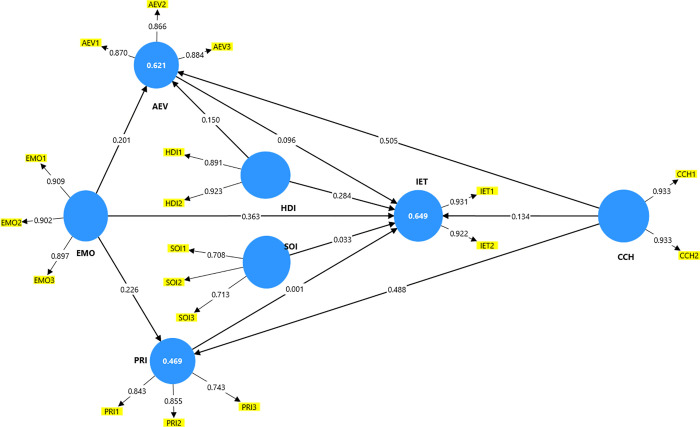
Measurement model results.

Fig 2 illustrates the results of the measurement model, including Cronbach’s alpha, composite reliability, average variance extracted (AVE), and discriminant validity.

Cronbach’s alpha of the indicators is greater than 0.7, which implies that they are valid [146]. Table 4 lists the loading values for each indicator. The reliability of the constructs was analyzed using Composite Reliability and Cronbach’s alpha, with a value greater than 0.7, which is acceptable [147]. Table 5 shows that each construct has satisfactory internal consistency and reliability, with values greater than 0.7. For each construct, convergent validity was analyzed using the average variance extracted (AVE); when the value was greater than 0.5, it was considered acceptable [148].

**Table 4:** Loadings of each indicator.

	AEV	CCH	EMO	HDI	IET	PRI	SOI
AEV1	0.870						
AEV2	0.866						
AEV3	0.884						
CCH1		0.933					
CCH2		0.933					
EMO1			0.909				
EMO2			0.902				
EMO3			0.897				
HDI1				0.891			
HDI2				0.923			
IET1					0.931		
IET2					0.922		
PRI1						0.843	
PRI2						0.855	
PRI3						0.743	
SOI1							0.708
SOI2							0.897
SOI3							0.713

**Table 5:** Reliability and convergent validity of the constructs.

	Cronbach’s Alpha	Composite Reliability	Average Variance Extracted (AVE)
AEV	0.844	0.906	0.763
CCH	0.852	0.931	0.871
EMO	0.886	0.930	0.815
HDI	0.786	0.903	0.822
IET	0.835	0.924	0.858
PRI	0.753	0.855	0.664
SOI	0.716	0.819	0.605

Discriminant validity analysis was conducted based on Fornell and Larcker [148], who determined whether the value of the square root of AVE was greater than the inter-construct correlations. For the present model, this condition was 100% satisfied in all cases, as indicated in Table 6. A cross-loading check was also performed to strengthen discriminant validity analysis. For the model, it was validated that each indicator correlated more with its own latent variable than with others, as indicated in Table 7.

**Table 6:** Fornell-Larcker’s criterion test.

	AEV	CCH	EMO	HDI	IET	PRI	SOI
AEV	0.873						
CCH	0.765	0.933					
EMO	0.712	0.811	0.903				
HDI	0.615	0.651	0.681	0.907			
IET	0.649	0.706	0.756	0.697	0.927		
PRI	0.703	0.672	0.622	0.570	0.565	0.815	
SOI	0.501	0.542	0.639	0.560	0.546	0.552	0.778

**Table 7:** Cross-loadings.

	AEV	CCH	EMO	HDI	IET	PRI	SOI
AEV1	0.870	0.628	0.605	0.523	0.563	0.567	0.423
AEV2	0.866	0.670	0.602	0.519	0.552	0.618	0.422
AEV3	0.884	0.703	0.656	0.567	0.585	0.655	0.466
CCH1	0.719	0.933	0.773	0.584	0.663	0.615	0.490
CCH2	0.709	0.933	0.741	0.631	0.654	0.639	0.520
EMO1	0.669	0.769	0.909	0.620	0.711	0.549	0.538
EMO2	0.640	0.698	0.902	0.629	0.668	0.595	0.618
EMO3	0.618	0.728	0.897	0.594	0.668	0.541	0.576
HDI1	0.505	0.531	0.562	0.891	0.579	0.471	0.465
HDI2	0.603	0.642	0.666	0.923	0.679	0.557	0.546
IET1	0.612	0.670	0.715	0.680	0.931	0.537	0.531
IET2	0.591	0.637	0.685	0.610	0.922	0.510	0.480
PRI1	0.698	0.670	0.599	0.514	0.550	0.843	0.415
PRI2	0.563	0.524	0.500	0.477	0.444	0.855	0.503
PRI3	0.401	0.396	0.384	0.383	0.353	0.743	0.449
SOI1	0.228	0.225	0.273	0.304	0.245	0.362	0.708
SOI2	0.553	0.612	0.715	0.575	0.603	0.527	0.897
SOI3	0.239	0.246	0.299	0.322	0.264	0.347	0.713

The measurement model is ultimately valid, implying that the instrument is statistically valid and reliable, and that the theory is supported [149].

### 4.2. Structural Model

It was executed based on the analysis of the weight and magnitude of the relationships between variables; this involved the R^2^ analysis, f^2^ effect, standardized path coefficients β, and bootstrapping analysis. R^2^ defines the predictive power of the model with values above 0.67, 0.33, and 0.19. denoted substantial, moderate, and weak, respectively [145]. Table 8 shows the values that ensure the percentage of variability of the construct, thus confirming the predictive characteristics of the model.

**Table 8:** R^2^of dependent variables.

	R Square	R Square Adjusted	Level
AEV	0.621	0.620	Moderate
IET	0.649	0.647	Moderate
PRI	0.469	0.468	Moderate

f^2^ determines the impact of a variable on the dependent construct. f^2^ > 0.35, it implies a large effect size; 0.15 < f^2^ ≤ 0.35 implies a medium effect; and 0.02 < f^2^ ≤ 0.15 implies a small effect. Table 9 presents these details.

Table 9 highlights that consumer characteristics have a medium effect, while emotions and hyperbolic discounting have a small effect on attitude towards electric vehicles. On the other hand, attitude towards electric vehicles, consumer characteristics, emotions, hyperbolic discounting, perceived risk, and social influence have a small effect on the intention to use electric transportation. Additionally, consumer characteristics had a medium effect, whereas emotions had a small effect on perceived risk.

**Table 9:** Effect Size f^2^.

	AEV	Category	IET	Category	PRI	Category
AEV			0.090	Small		
CCH	0.218	Medium	0.130	Small	0.154	Medium
EMO	0.032	Small	0.098	Small	0.033	Small
HDI	0.030	Small	0.108	Small		
PRI			0.004	Small		
SOI			0.003	Small		

### 4.3. Hypothesis testing

**Fig 3:**
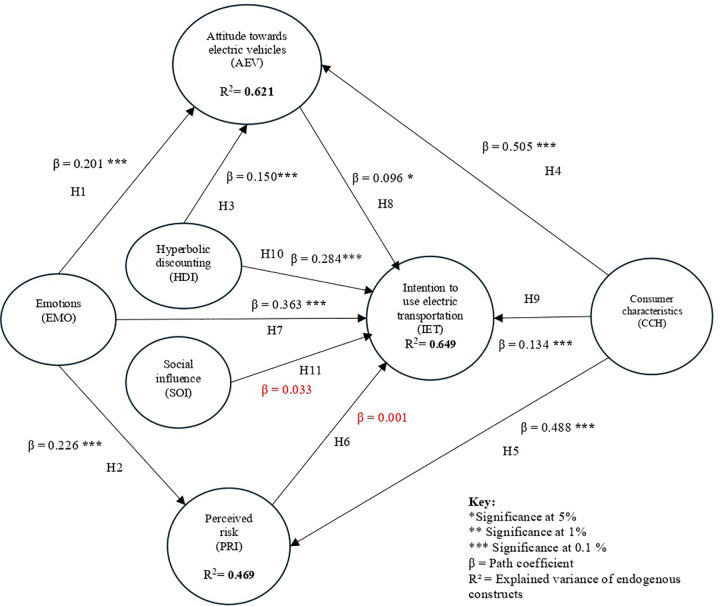
Result of the bootstrapping.

In Fig 3, hypothesis testing in Smart PLS is primarily carried out through the bootstrapping technique, a resampling method that allows for evaluating the robustness and significance of the structural model coefficients [150]. In general, a t-value greater than 1.96 indicates that the relationship between constructs is statistically significant at the 5% significance level (p < 0.05) [145,151].

Additionally, Smart PLS calculates confidence intervals from the bootstrapping process, providing an additional assessment of the accuracy of the estimates. It is important to note that hypothesis testing is not solely based on the t-value but also on the analysis of standardized coefficients (β), the standard error, and p-values, which provide a clearer and more detailed view of the relationships between the constructs in the model [137]. Table 10 presents the relationships between the constructs, including standardized beta coefficients, standard errors, t-values, significance levels, and the acceptance or rejection of each hypothesis

**Table 10:** Summary of results for the structural model.

	β	Standard Error	t – student	p values	Significant Level	Results
H1: EMO - > AEV	0.201	0.046	4.325	0.000	***	Accepted
H2: EMO - > PRI	0.226	0.044	5.123	0.000	***	Accepted
H3: HDI - > AEV	0.150	0.033	4.498	0.000	***	Accepted
H4: CCH - > AEV	0.505	0.049	10.373	0.000	***	Accepted
H5: CCH - > PRI	0.488	0.043	11.240	0.000	***	Accepted
H6: PRI - > IET	0.001	0.038	0.038	0.969		Rejected
H7: EMO - > IET	0.363	0.043	8.416	0.000	***	Accepted
H8: AEV - > IET	0.096	0.040	2.407	0.016	*	Accepted
H9: CCH - > IET	0.134	0.048	2.789	0.005	***	Accepted
H10: HDI - > IET	0.284	0.038	7.516	0.000	***	Accepted
H11: SOI - > IET	0.033	0.029	1.141	0.254		Rejected

*** p < 0.001; ** p < 0.01; * p < 0.05.

To ensure the validity of the results, the sample size of this study met the requirements established for PLS-SEM. While the method recommends a minimum of 50 observations, 1,158 valid surveys were applied, ensuring adequate statistical power and minimizing the risk of type II errors [137]. Regarding measurement errors, all indicators had a Cronbach’s alpha greater than 0.7 (see Table 5), indicating high reliability. Additionally, the factor loadings are greater than 0.7 (see Table 4), and the discriminant validity analysis confirms that the constructs measure distinct dimensions without excessive correlations (see Tables 6 and 7).

Regarding model configurations, the relationships between the constructs were theoretically supported and the constructs were correctly defined and aligned with their theoretical dimensions. Additionally, multicollinearity was checked, with VIF values ranging from 1.875 to 3.884 (see Table 11), well below the critical threshold of 5, indicating that there are no multicollinearity issues that affect the stability of the model [150]. Furthermore, the significance of all the structural paths was confirmed.

**Table 11:** Collinearity statistics (VIF) inner model.

	VIF
AEV - > IET	3.010
CCH - > AEV	3.086
CCH - > IET	3.884
CCH - > PRI	2.922
EMO - > AEV	3.315
EMO - > IET	3.819
EMO - > PRI	2.922
HDI - > AEV	1.968
HDI - > IET	2.137
PRI - > IET	2.342
SOI - > IET	1.875

Despite these measures, the authors acknowledge that self-selection may have affected the results. Therefore, they suggest that future research implement additional strategies, such as probabilistic sampling or surveys targeting a more representative sample, to improve the validity and generalizability of the findings.

Additionally, regarding the exploration of other factors, we recognize the importance of including moderating or mediating variables in the analysis. Although this aspect was not addressed in the present study, we recommend that future research incorporate moderating effects analysis (Multigroup Analysis) and more complex models to assess nonlinear relationships.

In this study, the following hypotheses were tested: The first hypothesis suggests that emotions (EMO) positively influence attitudes towards electric vehicles (AEV). Table 10 highlights the positive and strong influence of EMO on AEV (β = 0.201, t-value = 4.325, p < 0.001), thus supporting this hypothesis.

Hypothesis two suggests that emotions (EMO) influence perceived risk (PRI). Table 10 highlights the positive and significant influence of EMO on PRI (β = 0.226, t-value = 5.123, p < 0.001), thus, supporting this hypothesis.

Hypothesis three suggests that hyperbolic discounting (HDI) influences attitudes towards electric vehicles (AEV). Table 10 highlights the positive and significant influence of HDI on AEV (β = 0.150, t-value = 4.498, p < 0.001), thus, supporting this hypothesis.

Hypothesis four suggests that the characteristics of the consumer (CCH) influence their attitudes towards electric vehicles (AEV). Table 10 highlights a positive and significant influence of CCH on AEV (β = 0.505, t-value = 10.373, p < 0.001), the hypothesis is supported.

Hypothesis five suggests that consumer characteristics (CCH) influence perceived risk (PRI). Table 10 highlights the positive influence of CCH on PRI (β = 0.488, t-value = 11.240, p < 0.001), thus, supporting this hypothesis.

Hypothesis six suggests that perceived risk (PRI) does not influence intention to use electric transportation (IET). Table 10 highlights the non-significant influence of PRI on EIT (β = 0.001, t-value = 0.038). The p-value is not significant, so the hypothesis is rejected.

Hypothesis seven suggests that emotions (EMO) influence intention to use transportation (IET). Table 10 highlights a positive and significant influence of EMO on IET (β = 0.363, t-value = 8.416, p < 0.001), the hypothesis is supported.

Hypothesis eight suggests that attitude towards electric vehicles (AEV) influences the intention to use electric transportation (IET). Table 10 highlights a positive and significant influence of AEV on EIT (β = 0.096, t-value = 2.407, p < 0.05), the hypothesis is supported.

Hypothesis nine suggests Consumer characteristics (CCH) positively influence intention to use electric transportation (IET). Table 10 highlights a positive and significant influence of CCH on EIT (β = 0.134, t-value = 2.789, p < 0.05), the hypothesis is supported.

Hypothesis ten suggests that hyperbolic discounting (HDI) positively influences the intention to use electric transportation (IET). Table 10 highlights a positive and significant influence of HDI on EIT (β = 0.284, t-value = 7.516, p < 0.001), the hypothesis is supported.

Hypothesis eleven suggested that social influence (SOI) positively influences intention to use electric transportation (IET). Table 10 shows that SOI does not influence EIT (β = 0.033, t-value = 1.141, p is not significant); therefore, the hypothesis is rejected.

### 4.4 Model Fit Indicators

The overall model fit was evaluated using indicators recommended for PLS-SEM. The SRMR (Standardized Root Mean Square Residual) reflects the average discrepancy between observed and estimated correlations, with values below 0.08 considered acceptable [152]. The metrics d_ULS (Unweighted Least Squares discrepancy) and d_G (Geodesic discrepancy) represent discrepancies between the model and the data under different criteria and are interpreted by comparing their values with bootstrap distributions, as no fixed cutoff points exist [153]. The Chi-square statistic complements the fit assessment, although it is sensitive to sample size [154]. Finally, the NFI compares the model with a null model, with values above 0.90 considered acceptable [155].Together, these indicators provide a robust evaluation of the quality and validity of the model [156].

Table 12 presents the global fit of the model using indicators recommended for PLS-SEM, including SRMR, d_ULS, d_G, Chi-square, and NFI. The SRMR values were 0.082 for the saturated model and 0.089 for the estimated model, indicating an acceptable fit between the observed and estimated covariance matrices. The d_ULS values (1.151 and 1.349) and d_G values (0.440 and 0.483) suggest moderate discrepancies, typical in PLS-SEM models. The Chi-square increased from 3245.621 to 3437.464 in the estimated model, reflecting a slight loss of fit compared to the saturated model. Meanwhile, the NFI for the estimated model was 0.758, indicating a moderate fit relative to a null model. Overall, these results show that the structural model presents an acceptable fit, sufficient for exploratory and predictive analyses. However, the NFI suggests that further improvements could be explored, for example, by refining weak indicators or relationships to strengthen the overall model fit.

**Table 12:** Global fit indicators of the PLS-SEM structural model.

	Saturated model	Estimated model
SRMR	0.082	0.089
d_ULS	1.151	1.349
d_G	0.440	0.483
Chi-square	3245.621	3437.464
NFI	0.771	0.758

## 5. Discussion

H1: Hypothesis one suggests that the influence and positive impact of EMO on AEV is, thus, important to consider the emotional component in these relationships. Emotions play a fundamental role in the decision to purchase an EV; thus, recent studies have revealed that anticipated positive emotions, especially those related to environmental impact and governmental support policies, have a significant effect on favorable attitudes towards these vehicles [157].

Emotions are internal states that influence behavior; emotions are interconnected with motivations, and motivation is defined as the attraction to positive emotions and avoidance of negative emotions [158]. The interaction between emotional factors demonstrates the complexity of consumer attitudes toward EV adoption. While emotions related to enjoyment and aesthetic appreciation are important, it is reflective emotions rooted in the cognitive evaluation of sustainability and profitability that primarily shape positive attitudes and motivate EV adoption [62,159].

H2: Hypothesis two suggests a positive influence of EMO on PRI since emotions play an important role in the evaluation of risks associated with the purchase of EVs. Shu et al. [160] suggested that negative emotions, especially those related to uncertainty regarding charging and maintenance times, significantly increase the perception of risk. However, positive driving experiences may mitigate this perception. However, the relationship between emotions and risk is complex because both positive and negative anticipated emotions influence use intention. In conclusion, emotions act as filters through which consumers evaluate EV risks. Understanding this process is essential for designing more effective communication strategies.

Reflective emotions, which encompass cognitive evaluations of a vehicle’s environmental benefits, economic viability, and social acceptance, can influence the assessment of perceived risk. Therefore, positive reflective emotions surrounding environmental sustainability can shift attention from risks to benefits, increasing the likelihood of EV adoption [76]. Emotions such as hope and enthusiasm for the future of sustainable transportation could decrease perceived risks by fostering a spirit of adventure and a willingness to embrace change. Those with a positive attitude toward sustainability may be more likely to perceive the risks associated with EVs as manageable or acceptable. In conclusion, emotions act as filters through which consumers evaluate the risks associated with EVs. Understanding this process is essential for designing more effective communication strategies [60].

H3: Hypothesis three suggests that HDI positively influences AEV. Hyperbolic discounting, understood as the preference for small, immediate rewards over larger, long-term rewards, significantly influences consumers’ attitudes toward EVs [88]; individuals high in hyperbolic discounting are more likely to purchase EVs if offered immediate financial incentives, such as discounts or rebates [111]. However, the effectiveness of financial incentives may vary according to other factors, such as environmental policies and socioeconomic characteristics of consumers [77]. Despite this, the evidence suggests that hyperbolic discounting is an important psychological factor to consider when designing strategies to promote the adoption of EVs.

Hyperbolic discounting exerts a significant influence on the formation of consumer attitudes toward EVs, distorting the rational evaluation of costs and benefits through a disproportionate weighting of immediate rewards at the expense of future rewards. Recognizing this cognitive bias, stakeholders, including government entities and marketing professionals, can implement strategies designed to optimize the perceived long-term value inherent in EVs, with the potential to induce more favorable attitudes and, consequently, increase adoption rates of EV technology [56,58,161]. In summary, this study provides substantial evidence to support the hypothesis that hyperbolic discounting significantly influences consumer attitudes towards EVs, emphasizing the importance of understanding this bias when aiming to promote EV adoption.

H4: Hypothesis four suggests that the characteristics of the consumer (CCH) positively influence attitudes towards electric vehicles (AEV). Individual consumer characteristics play a key role in the adoption of EVs. Sociodemographic, psychographic, and lifestyle factors significantly influence attitudes and purchase decisions [72]. Thus, consumers with a higher income, education, and environmental awareness tend to show a greater predisposition towards EVs. These individuals value aspects such as sustainability, innovation, and the social status associated with technology [162]. Demographic profiles show significant differences in environmental concerns, which are crucial for promoting EV adoption. Individuals who prioritize environmental protection tend to have more favorable views of EVs. Overall, demographic factors interact with psychological and situational variables to shape consumers’ intentions and attitudes towards EVs [58,159].

H5: The consumer characteristic hypothesis (CCH) positively influences perceived risk (PRI) and complies with the study, as factors such as income, age, and educational level of consumers have a positive impact on how they perceive risk and their purchase decisions, especially in the electric and e-commerce markets. This is because consumers’ perceptions of the relationship between risk and benefit play an important role in EV acceptance [72]. As Table 10 shows, the p-values are highly significant (less than 0.05); therefore, this hypothesis is positively accepted within the model. Potential users of EVs are attentive to how safety, performance, and compatibility standards affect the perception of risk influenced by aspects such as product quality and consumer confidence, which induces their willingness to use renewable-energy vehicles [65]. Prospective buyers’ attention to safety, performance, and compatibility standards reflects legitimate concern for the investment they are about to make. Product quality and brand confidence play crucial roles in reducing risk perceptions. When consumers feel that an EV meets high standards, they are more likely to feel comfortable and motivated to use it.

The results of the present study are consistent with previous research conducted in countries such as Norway and Germany, where sociodemographic factors such as age, income, and educational level have been shown to significantly influence risk perception and EV adoption [163]. In this context, younger consumers with higher levels of income, education, self-efficacy, and an inclination toward innovation tend to perceive less risk and adopt these technologies more easily [164]. Moreover, effective public policies play a fundamental role in reducing consumer concerns and promoting acceptance [165].

H6: Regarding the hypothesis that perceived risk (PRI) negatively influences intention to use electric transport (IET), Table 10 shows that the p-value is 0.969, meaning that the result is higher than 0.05. This shows that these variables were not significant and were therefore rejected in this study. The purchase of an EV implies high costs and significant risks, which may lead consumers to not make this decision without careful evaluation and exhaustive reflection on available options [73]. Factors that increase consumers’ perception of risk when purchasing EVs include limited battery capacity, a lack of adequate charging infrastructure, and associated costs [166]. Some elements that contribute to the increased risk perceived by consumers when purchasing an EV include limited battery capacity, lack of adequate charging infrastructure, and associated costs, which are critical because they directly affect the user experience and desirability of owning an EV. The associated costs, which may include the initial price of the vehicle, maintenance costs, and cost of installing a charging station at home, can influence the perceived value and financial viability of choosing this type of vehicle [73]. The research described above is related to the results obtained in the study, demonstrating that university students perceive risk in their use decisions when acquiring an electric transportation service. Therefore, it is necessary to note that both physical safety and performance risks have a negative impact on the perception of value, which may decrease consumers’ willingness to use electric vehicles.

In countries such as India, it has been shown that perceived risk negatively affects the intention to adopt electric vehicles, suggesting that risk perception is a common factor influencing technology adoption worldwide [167]. In Sri Lanka, a negative relationship between perceived risk and intention to adopt EVs has also been identified [168]. However, the results obtained in the present study do not align with these findings, as the hypothesis was rejected, indicating that, in the analyzed context, perceived risk does not have a significant effect on the intention to use electric transportation.

H7: The hypothesis that emotions (EMO) positively influence intention to use electric transportation (IET) indicates that emotions play an important role in consumer behavior, and that positive emotions can increase intention to use electric transportation, mediated by the perception of value [169]. Favorable emotional attitudes towards EVs are related to the intention to adopt EVs, suggesting that emotions have a considerable impact on the decision to use electric transportation, highlighting the importance of emotions in the consumer decision-making process. Inducing positive emotions such as satisfaction and hope associated with EVs, may motivate consumers to consider this option viable and attractive [170]. According to the above statements, it is evident that the p-values are higher than 0.05; therefore, this hypothesis is accepted. Emotions and attitudes towards EVs are determinants and important in the intention to use them, which indicates that the emotional responses of the respondents are positive, leading to an improvement in the intention to use electric transportation. This study focuses on the emotional appeal of EVs and suggests that positive emotions can elevate consumers’ intention to opt for electric transportation.

It has been observed in the Chinese market that emotions, particularly tastes and preferences, positively influence the intention to use electric transportation [171]. Similarly, anticipated positive emotions play a key role in the intention to use electric transportation in India, Turkey, and Brazil. These emotional connections could also have a favorable impact on the intention to use electric transportation in other emerging markets [157,172,173]. These findings were consistent with the results of the present study.

H8: Attitude towards electric vehicles (AEV) positively influences the intention to use electric transport (ETI). A favorable attitude significantly increases consumers’ interest in adopting EVs, which in turn has a positive effect on their intention to use electric transport [102]. Attitudes towards EVs have a considerable impact on the intention to adopt them, as favorable ratings increase the likelihood that people will opt for electric transportation alternatives. Consumers with a favorable view are more likely to be motivated to consider this option over more traditional alternatives [106]. From the contribution of this research, it is evident that the result of this hypothesis is acceptable, where the p-value is less than 0.05, thus highlighting that university students’ attitudes directly influence their use of electric transportation.

This study shows that attitude has a significant influence on consumers’ willingness to adopt EVs, suggesting that favorable beliefs about EVs increase their intention to use electric transportation. When consumers develop a favorable perception of these vehicles, they are more motivated to choose electric transportation options rather than relying on traditional alternatives. This phenomenon suggests that companies and policymakers should focus on improving the image of electric vehicles by highlighting their benefits such as sustainability, fuel economy, and low maintenance.

Attitude has a significant impact on consumers’ intention to adopt electric transportation in Spain, suggesting that this factor may similarly influence other contexts [174]. Likewise, in countries such as China and Malaysia, it has been demonstrated that a positive attitude significantly enhances consumers’ willingness to adopt EVs, indicating that fostering favorable perceptions could strengthen the intention to use electric transportation globally [175,176]. In this regard, the results obtained in the present study are consistent with these findings, confirming that attitude is a key determinant in the intention to use electric transportation.

H9: Hypothesis nine states that Consumer Characteristics (CCH) positively influence the intention to use electric transportation (IET); Table 10 highlights the positive and significant influence of CCH on IET (β = 0.134, t-value = 2.789, p < 0.05). In this study, it was shown that students associate the use of an EV with a positive image of the university. This characteristic aligns with Silva and Sousa [177] and Lampo [178]. It was also shown that students associate possession of an electric vehicle with the university’s environmental responsibility. In a study conducted in China Sovacool, Abrahamse, Zhang, & Ren, [179] demonstrated that environmental attributes play a significant role in people’s decision to adopt EVs. Similarly, in Canada, individuals with pro-environmental lifestyles are more likely to use EVs [180]. These aspects were positively evaluated by students regarding the university’s use of EVs.

Likewise, in Malaysia, Choo et al. [181] found that people with environmental awareness are positively associated with the intention to use EVs. Degirmenci and Breitner [182] also demonstrated that the environmental behavior of EVs is a predictor of consumers adopt intention, even stronger than price value and range confidence.

H10: Hypothesis ten assumes that Hyperbolic Discounting (HDI) has a positive influence on the intention to use electric transportation (IET); Table 10 has a positive and significant influence on IET (β = 0.284, t-value = 7.516, p < 0.000). This study showed that university students were willing to accept small but immediate discounts that allowed them to use electric buses, rather than larger rewards in the future. This result is consistent with the findings of Muehlegger, and Rapson [183] and a study by New Zealand [184]. Additionally, university students are willing to pay a higher price for the use of electric vehicles, provided that financial incentives are offered. This is consistent with Deepak et al. [185], who found in a study conducted in India that financial incentives have a positive moderating effect on consumer attitudes toward the intention to adopt EVs. This is also in line with Sierzchula et al. [110], who conducted a study using data from 30 developed countries, including Europe, Japan, Australia, Nordic countries, and China, and with Clinton and Steinberg [186] in a study in the United States. This is also consistent with the findings of Adnan et al. [187] in a study conducted in Malaysia, where hyperbolic discounting moderated the relationship between behavioral intention and the adoption of electric transport.

H11: Hypothesis 11 posits that Social Influence (SOI) is positively related to the Intention to Purchase Electric Vehicles (IET); Table 10 highlights the positive but insignificant influence of SOI on IET (β = 0.033, t-value = 1.141, p > 0.05). In this study, the hypothesis was not supported, indicating that the opinions of friends, family, peers, professors, and society in general do not influence university students’ decisions to adopt EVs. Several studies have confirmed these results. For example, Hoang et al. [188] and Jansson et al. [189].

This result draws the attention of researchers, since, in general, young people and students tend to have a positive attitude toward new technologies and environmental protection. This is demonstrated by a study conducted by TT N Nho and N T V [190] with students in Ho Chi Minh City, Vietnam, where environmental attitude, social value, and attitude toward green consumption positively influence the intention to adopt EVs. Along these lines, a study by Mikhail [191] with university students in Moscow showed how and to what extent environmental beliefs contribute to the use of eco-friendly modes of transportation in this case, urban electric buses which received high acceptance among the study population.

Student adoption of electric buses is increasing in developing regions, driven by urbanization and environmental awareness [192,193]. Adoption is faster in Asia due to incentives, advanced technology, and charging networks, slower in Africa due to structural and financial barriers, and intermediate in Latin America, including Ecuador, Peru, and Colombia [194,195]. These findings suggest that governments and universities should implement targeted strategies, such as point-based incentives for electric bus use, accessible charging infrastructure, sustainable mobility programs, and awareness campaigns for students. The lack of significance of social influence and perceived risk indicates that students make more utilitarian and environmentally conscious decisions, highlighting electric transportation as a factor for economic, social, and environmental development.

## 6. Research contributions and implications

### 6.1. Theoretical contribution

A key theoretical contribution of this study is the integration of technology adoption models (TAM, TPB, UTAUT) to explain the adoption of electric buses by identifying key predictive factors, such as emotions, attitudes, and perceived risk. Analyzing the proposed hypotheses and validating the positive influence of variables such as emotions and consumer characteristics on the intention to use highlights the fundamental role of these factors in the adoption of sustainable technologies. The rejection of the hypotheses, which suggests that perceived risk and social influence are not as decisive as previously thought, refine our understanding of the real drivers behind the adoption of electric transportation.

### 6.2. Practical contribution

A practical application of this study is to design awareness and promotion campaigns for the use of electric buses, focusing on the factors that most influence their adoption. For example, concerns about perceived risk can be reduced by providing clear information on the safety and benefits of EVs. Additionally, integrating economic incentives that mitigate hyperbolic discounting, such as offering immediate rewards for using a service, could increase intention to use it. These campaigns could also leverage social influence by highlighting the use of electric buses by student leaders and influential social groups.

For local governments, the incorporation of electric buses into the fleet is suggested to promote environmental care and generate targeted awareness among university students to encourage their use as a daily means of transportation. Likewise, the inclusion of charging infrastructure is recommended, promoting not only electric public transportation but also private electric transportation. Thus, a moral obligation is fostered among citizens to reduce the use of fossil fuel vehicles.

### 6.3. Research limitations

The data were collected through coordination led by the Universidad Católica de Cuenca, Ecuador. It is important to clarify that this university does not have campuses in Colombia or Peru; however, due to efforts made in Ecuador, collaboration was successfully established with universities in those countries to contribute to data collection.

It is important to note that data collection was conducted from April to July, a period that corresponds to a specific academic semester in the participating universities. Seasonal or semester-related factors such as academic workload, on-campus presence, weather conditions, and transportation availability may influence students’ mobility needs and attitudes toward electric transportation. Consequently, perceptions captured during this period may differ from those in other semesters or seasons, representing a contextual limitation of the study.

This study may not have considered external factors that influence the adoption of electric buses, such as government policies, economic incentives, or existing transportation infrastructure in the region. Attitudes and perceptions of risk may vary significantly among the demographic and cultural groups, which may not have been fully represented in the sample. Although the intentions to use were analyzed, this does not guarantee that they will translate into actual adoption behaviors, which could limit the practical application of the findings.

### 6.4. Future projects

For future research, it is recommended to consider using CB-SEM to validate theoretical models, Bayesian SEM for more flexible estimations, or Nonlinear SEM to capture more complex relationships. This approach will provide a comprehensive and robust understanding of electric bus adoption in emerging countries and offer methodological guidance for subsequent studies addressing nonlinear and group-specific effects.

Growing concerns about climate change and sustainability have led to increased interest in electric vehicles, especially among younger generations. As future professionals and citizens, college students play a crucial role in adopting sustainable transportation alternatives. For future studies, a deeper understanding of the factors influencing the intention to use electric transportation services is required. The intention of college students to adopt electric transportation is a promising topic for future research. Understanding the factors that influence their decision to adopt electric vehicles can not only help manufacturers and potential consumers but also contribute to a broader cultural shift toward more sustainable transportation. On the other hand, future studies could further explore the differences between adoption and acquisition/purchase.

Future studies should examine the extent to which academic-semester dynamics and seasonal variations influence students’ adoption intentions and mobility preferences. Moreover, it would be highly valuable to analyze mediating variables in the conversion from intention to behavior such as: perceived behavioral control [45], habit formation [196], perceived risk [197], and contextual constraints [198] to better understand the intention–behavior gap in sustainable transportation adoption. Investigating these mediators, potentially through longitudinal or experimental designs, could provide deeper insights into the mechanisms that facilitate or hinder the transition from intention to actual use of electric transportation services.

## 7. Conclusions

This study demonstrated the feasibility of introducing electric buses for student transportation at universities in Ecuador, Colombia, and Peru, highlighting their potential as sustainable alternatives to fossil fuels. Through a technology adoption model, key factors influencing the intention to use, such as attitudes and risk perceptions, were identified. These findings emphasize the importance of implementing comprehensive strategies to promote the adoption of sustainable transportation solutions in student communities

The proposed hypotheses revealed a complex interplay between factors influencing the intention to use electric vehicles (EVs). Emotions significantly drive attitudes toward EVs and perceived risks, whereas hyperbolic discounting indicates that consumers may prioritize immediate benefits over long-term gains. Individual consumer characteristics also shape perceptions and affect attitudes and risk assessment. Perceived risk negatively affects the intention to adopt EVs, highlighting the need to address consumer concerns. Positive attitudes and social influences are crucial for encouraging EV usage and underlining the importance of effective messaging and community support. Overall, a multifaceted approach is essential for promoting electric vehicle adoption, considering the emotional, psychological, and social factors.

University students’ adoption of electric bus services by university students is influenced by their environmental awareness, economic benefits, and institutional support. As universities continue to promote sustainable transportation and provide the necessary infrastructure and education, it is likely that students’ adoption of EVs will continue to grow, contributing to broader environmental and economic benefits.

University students view electric transport as a more sustainable option because of its modern nature, technology, safety, and comfort. It is crucial for higher education institutions to raise environmental awareness and promote EV adoption to address environmental challenges. By highlighting the benefits of electric transportation and addressing safety and regulatory concerns, universities can encourage the use of green transportation options, thereby contributing to a more sustainable future.

## Supporting information

S1 Appendix Questionnaire items development based on literature [199–202]. (DOCX)
