# Supplementary material for: Intention to adopt electric transportation services by university students in emerging countries
Source: PLoS One. 2026 Jan 27;21(1):e0341736. doi: 10.1371/journal.pone.0341736 (PMC12843562; doi:10.1371/journal.pone.0341736)
Supplement: S1 Appendix — (DOCX) [file pone.0341736.s001.docx]

| Variable: Attitude towards electric vehicles (AEV)  Source: (199) (70) | |
| --- | --- |
| AEV1 | In general, do you have a positive attitude towards electric vehicles? |
| AEV2 | Do you believe that electric vehicles are beneficial for the environment? |
| AEV3 | Do you consider electric vehicles to be an attractive option for staff transportation? |
| **Variable:** Perceived risk (PRI)  **Source**: (68) | |
| PRI1 | Do you feel that electric vehicles are less harmful to the environment than internal combustion vehicles? |
| PRI2 | Do you think electric vehicles are more reliable than internal combustion engine vehicles? |
| PRI3 | Do you perceive that electric vehicles have a lower risk of experiencing mechanical failures? |
| **Variable:** Consumer characteristics (CCH)  **Source:** (72) (91) | |
| CCH1 | Do you think that the electric vehicle transportation service provided by the university would reflect a positive image? |
| CCH2 | Do you associate owning an electric vehicle with the University’s environmental responsibility? |
| **Variable:** Emotions (EMO)  **Source:** (82) (200) | |
| EMO1 | Are you excited about the idea of having an electric vehicle service in the university? |
| EMO2 | The idea of traveling in an electric vehicle makes you feel good about yourself. |
| EMO3 | Are you curious about experiencing travel in an electric vehicle? |
| **Variable:** Social influence (SOI)  **Source:** (201) (80). | |
| SOI1 | Do the opinions of your friends and family influence your decision to use an electric vehicle service? |
| SOI2 | If your peers and teachers used the electric vehicle service, would you be more likely to consider using that service? |
| SOI3 | Does the influence of society in general affect your perception of electric vehicles? |
| **Variable:** Hyperbolic discounting (HDI)  **Source:** (202) | |
| HDI1 | Would you be willing to pay a higher price for using an electric vehicle transport service compared to one that uses hydrocarbons? |
| HDI2 | Do you value the long-term benefits of using an electric vehicle service more than the immediate benefits? |
| **Variable:** Intention to use electric transportation (IET)  **Source**: (97) (169) | |
| IET1 | Do you think an electric transportation service would be convenient for your daily commutes? |
| IET2 | Would you use the electric transportation service for your trips within the university campus? |
